# Diagnostic Value of Rapid Biophysical Profile in Comparison to Biophysical Profile in Pregnant Women with Insulin-Dependent Diabetes 

**Published:** 2019-12

**Authors:** Nasrin Soufizadeh, Fariba Farhadifar, Saghar Tamri, Sara Behafarid, Karim Sharifi, Sima Aslani, Mobin Naqshbandi

**Affiliations:** 1 Department of Obstetrics and Gynecology, Faculty of Medicine, Kurdistan University of Medical Sciences, Sanandaj, Iran; 2 Department of Radiology, Faculty of Medicine, Kurdistan University of Medical Sciences, Sanandaj, Iran; 3 Student Research Committee, Kurdistan University of Medical Sciences, Sanandaj, Iran; 4 Student Research Committee, Iran University of Medical Sciences, Tehran, Iran

**Keywords:** Rapid Biophysical Profile, Biophysical Profile, Embryo Health, Pregnant Women, Insulin-dependent Diabetes

## Abstract

**Objective:** Having a rapid and low cost diagnostic approach in assessment of fetal wellbeing is an important goal for prenatal care process. The aim of this study was to determine the diagnostic value of rapid biophysical profile **(**rBPP) in comparison to biophysical profile (BPP).

**Materials and methods:** In this study 142 pregnant women with insulin-dependent diabetes referred to Besat Hospital (Sanandaj, Iran) were evaluated in terms of fetal health. Age, gestational age and non-stress test (NST) data of patients were collected. The fetuses were evaluated using the standard BPP and selected rBPP methods. Sensitivity, specificity, positive predictive values (PPV) and negative predictive values (NPV) were calculated. The receiver operating characteristic (ROC) curve was plotted. The data were analyzed in Stata 14 software, using appropriate statistical analyses.

**Results:** The mean ± standard deviation (SD) of maternal age and gestational age of the studied subjects were 30.6 ± 6.3 and 35.6 ± 1.5 weeks, respectively. The frequency of normal cases were 126 (88.7%) in the BPP method and 121 (85.2%) in the rBPP method. The results showed that sensitivity, specificity, PPV and NPV of rBPP in this study were 56.2%, 90.5%, 42.8% and 94.2%, respectively. The area under the ROC curve was 73.3%. Pearson Test showed a significant correlation between scores obtained through BPP and rBPP methods (p < 0.001).

**Conclusion:** Considering the high profile of the sensitivity and PPV of the RBPP method compared to BPP, rBPP method has a better capacity to discriminate non-distressed fetuses from distress-exposed fetuses. It can also be used as a quick and easy method in crowded centers with limited evaluation tests, where not much skill is needed.

## Introduction

One of the essential components of the prenatal care process is the evaluation of fetal health, which aims to identify at-risk fetuses and prevent complications (e.g. fetal and infant mortality). The most common prenatal tests that check fetal health include the assessment of fetal movements, non-stress test (NST), oxytocin challenge test (OCT) and fetal biophysical profile (FBPP) (1-3). FBPP is a combination of NST and four embryonic ultrasound parameters that was first examined by Manning et al. (1985) (4, 5). BPP is one of the prenatal tests that is used to diagnose fetal complications. The BPP method evaluates the five factors of fetal movement: respiration, tonicity, fluid level, and NST. It is are liable test for assessing the fetal health in high-risk pregnancies with the highest accuracy and the lowest false positive rate (6, 7). Being approved by many researchers in the field of fetal care, the rBPP measures the amniotic fluid index using sound-provoked fetal movement (SPFM) test (3). Recently, vibroacoustic stimulation (VAS) has been proposed to reduce non-reactive cases and NST time. In addition, VAS method can awake the sleeping embryos (8). Previous studies reported a 50% reduction in non-reactive results and shorter test time for VAS. Reaction criteria in VAS method are similar to those in NST and are reliable as a spontaneous reaction (9, 10). Therefore, due to the limited financial and human resources, and increasing number of the women who getting pregnant despite having risk factors, it seems that VAS test can reduce mother's and physician's concerns, while detect high-risk fetuses better. Additionally, it is a cheap, easy and non-invasive method that saves time and money (11). The present study was conducted to evaluate the diagnostic value of rBPP in comparison with BPP in diagnosing fetal health in pregnant women with insulin-dependent diabetes over 34 weeks.

## Materials and methods

***Patients and gathering their demographic and clinical records: ***Totally 142 pregnant women with insulin-dependent diabetes over 34 weeks referred to Besat Hospital (Sanandaj, Iran) during 2017-2018, were participated in this study. The approval for this study was obtained from the Ethics Committee of Kurdistan University of Medical Science (IR.MUK.REC.1396.105). Informed consent was obtained from the subjects after explaining the method. Age, gestational age, and NST data were obtained from patients. 

***Performing BPP and rBPP methods: ***In BPP method, the condition of the fetus in the uterus is evaluated in terms of heart rate, fetal breathing status, fetal movements, fetal muscle tone (the ability of the fetus to bend the legs and hands and its physical response to collision) ,and the level of amniotic fluid, according to the criteria described in Table 1. Briefly, Scores from 8 to 10 indicate the fetal proper wellbeing. Scores 6 indicate that the fetus should be re-evaluated within next 12-24 hours. Scores 4 or lower indicate serious complications and further investigation is required (4, 12, 13). The rBPP method is also used to evaluate the fetus, according to Table 1. In this method, the amniotic fluid index (AFI) and fetal response to acoustic stimuli are evaluated. rBPP has 2 items, each item having 2 points. Briefly, to perform the method, Braun model German-made electric toothbrush was used at a frequency of 50-60 Hz for 3 seconds above the mother’s abdomen to stimulate the fetus. In normal mode, fetal movement was observed within 15 seconds after the stimulation. In the absence of fetal movement, the test was repeated up to 3 times. Abnormal test results were defined ≤ 6 score for BPP and ≤ 2 score for rBPP. Frequency, mean of the variables as well as sensitivity, specificity, Positive Predictive Values (PPV), and Negative Predictive Value (NPV) were calculated.

***Statistical analyses: ***TheReceiver Operating Characteristic (ROC) curve was plotted. Pearson correlation Test was used to calculate the correlation between the scores of the two methods. Data were analyzed using Stata 14 software. The significance level was considered to be p < 0.05.

## Results

The results indicated that the mean and standard deviation of maternal age was 30.6 ± 6.3 years, and the mean gestational age was 35.6 ± 1.5 weeks. In this study, the abundance of normal cases was 126 (88.7%) in the BPP method and 121 (85.2%) in the RBPP method. The results also showed that 113 (79.6%) of NST cases were reactive (Table 2). Sensitivity, specificity, PPV and NPV of rBPP in this study were 56.2%, 90.5%, 42.8% and 94.2%, respectively (Table 3).

**Figure 1:**
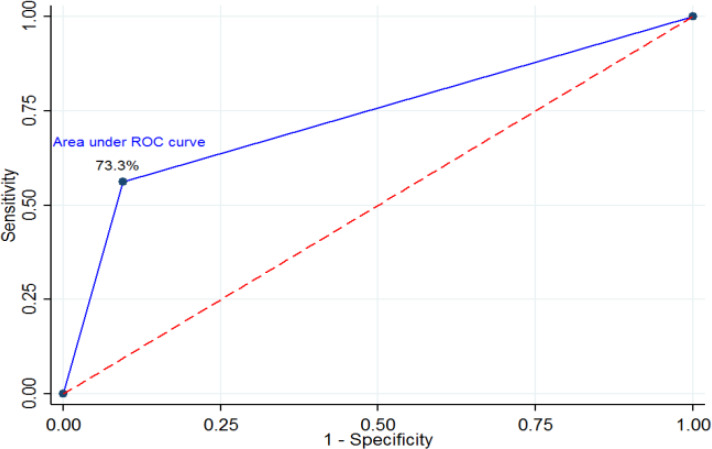
ROC curve for scores obtained through BPP and RBPP methods

The area below the ROC curve was 73.3% (Figure 1). Pearson test showed a significant correlation between scores obtained through BPP and RBPP methods (p < 0.001).

**Table 1:** Evaluation and scoring method in the BPP and RBPP methods

**Biophysical profile**				**Rapid Biophysical Profile**		
**Biophysical ** **variable**	**Normal** **(score = 2)**		**Abnormal** **(score = 0)**	**RBPP**	**Normal ** **(score = 2)**	**Abnormal ** **(score = 0)**
Fetal breathing movements (FBM)	One or more episodes of FBM > 30 sec in 30 min		Absent or no episode of FBM > 30 sec in 30 min	SPFM	Response	Non response
Gross body movements	Three or more discrete body/limb movements in 30 min (episodes of active continuous movement considered as single movement)		Two or less episodes of body/limb movements in 30 min	AFI	> 5 cm	≤ 5 cm
Fetal tone	One or more episodes of active extension with return to flexion of fetal limb(s) or trunk; opening and closing of hand considered normal tone		Either slow extension with return extension with return to flexion to partial flexion or movement of limb in full extension or absent fetal movement	Total (score)	4	0
Reactive fetal heart rate	Two or more episodes of acceleration of > 15 bpm and of > 15 sec associated with fetal movement in 20 min		Less than 2 episodes of acceleration of FHR or acceleration of < 15 bpm in 40 min			
Amniotic fluid volume	> 5 cm		≤ 5 cm			
Interpretation	Score = 8-10 Score = 6 Score = 0- 4	Normal fetus Fetal hypoxia is suspicious Fetal hypoxia			Score = 4 Normal fetus Score = 0-2 Fetal hypoxia	

**Table 2:** Demographic variables in pregnant women with insulin dependent diabetes

**Variables**	**Mean**	**SD**
Age	30.6	6.3
Age at Pregnancy	35.6	1.5
	**N**	**(%)**
BPP (Full biophysical profile)		
Score 8-10	100	70.4
Score 6	37	26.1
Score 0-4	5	3.5
RBPP(Rapid biophysical profile)		
Score 4	120	84.5
Score 0-2	22	15.5
NST (non-stress test)		
Reactive	93	15.3
Non-reactive	29	76.2
Apgar		
6	8	5.6
7	2	1.4
8	38	26.8
9	88	62
10	6	4.2

**Table 3:** Frequency distribution of normal and abnormal RBPP cases versus BPP in fetal health diagnosis and sensitivity, specificity, positive and negative predictive values of the RBPP method in fetal health diagnosis

**Method of ** **Diagnosis **		**BPP**		
		**Abnormal** **N (%)**	**Normal** **N (%)**	**Total**
RBPP	Abnormal	9 (56.2)	12 (9.5)	21 (14.8)
	Normal	7 (43.8)	114 (90.5)	121 (85.2)
	Total	16 (11.3)	126 (88.7)	142 (100)
		(95% Confidence Interval)		
	Sensitivity	56.2 (52.3-59.1)		
	Specificity	90.5 (86.4-93.6)		
	PPV	42.8 (39.7-46.3)		
	NPV	94.2 (90.4-97.6)		

## Discussion

RBPP is a non-invasive, fast and effort-less method, and, with a 56.2% sensitivity and a 90.5% specificity, it is a good test for the identification of healthy fetuses, because it has a higher specificity than sensitivity and positive predictive value, meaning that it can detect non-distressed fetuses from distressed fetuses. 

Prenatal care and fetal examinations are to reduce mortality and sustained neurological injuries, which has led to the development of various methods for assessing the fetal health. Today, the use of standard BPP, which includes the evaluation of the four current embryonic variables and a long-term fetal variable (AF), has significantly reduced perinatal mortality (5, 14). In this study, the diagnostic value of RBPP was compared to the BPP method. The findings showed that, with the BPP method, 100 of 142 cases (70.4%) had a score of 8 and more, and 37 of 142 cases (26.1%) scored 6. The evaluation of the fetuses by the RBPP method showed that 120 of 142 cases (84.5%) had a score of 4. In this study, abundance of non-reactive cases in the NST were about 20.4%. These results were comparable with previous studies. Baschat et al. (2006) reported that 48.2% of patients had a BPP score of >8 (15). On the other hand, Lotfalizadeh et al. (2014) reported that 83% of patients had a BPP score of >8 and 17% had a BPP score of <6. They also observed only 1.9% Non-reactive NST in the subjects and reactive NST included 98.1% of the subjects (16). The inconsistency between the results, is likely due to the type of patients, the number of studied subjects, and/or the different methodology.

Also, the results showed that sensitivity, specificity, PPV and NPV of the BPP method in this study were 56.2%, 90.5%, 42.8%, and 94.2%, respectively. In accordance with these results, Miller et al. (1996) showed that the percentage of false positive results in the BPP method was 60% and false negative results were 0.8% (17). In addition, Prabhu’s study showed that the specificity, sensitivity, and positive and negative predictive values of the rBPP test compared to BPP were 71.4%, 87.1%, 35.7%, and 96.8%, respectively. A study by Heidari et al. on high-risk pregnancies showed that fetal adverse events such as fetal distress, cesarean section due to fetal distress, low Apgar score, congenital anomalies, etc. in patients who had non-reactive test were more than those having reactive test; and sensitivity, specificity, and positive and negative predictive values of the test were estimated to be 33.3%, 91.9%, 58%, and 80%, respectively (18). In another study, Jamal et al. (2007) compared the results of BPP to Modified Biophysical Profile (MBP) tests and showed that the sensitivity, specificity and positive and negative predictive values were 87.5%, 96.9%, 93.3%, and 93.9% in the MBPP test and 84.6%, 97.4%, 91.7%, and 95% in the BPP test, respectively. They found out that sensitivity, specificity and negative predictive value were similar but positive predictive value was significantly different in the two tests (19). 

Important advantages of the rBPP method include rapid accomplishment and no need for NST. Acute hypoxia (SPFM) and chronic embryo hypoxia (using AFI) also can be examined in this method. RBPP method also reduces the referral to healthcare centers to perform BPP and does not require expensive ultrasound scanning with high diagnostic probability of acute and chronic fetal hypoxia.

## Conclusion

Given its 56.2% sensitivity and 90.5% specificity, rBPP is a reliable test for the identification of healthy fetuses. Importantly, it has a higher specificity than sensitivity and PPV; meaning that it can discriminate non-distressed fetuses from distressed fetuses. It can also be used as a quick and simple method in crowded centers with limited evaluation tests where easy-performing, rapid, and cheap diagnostic methods are highly demanded.
